# A Comparative Evaluation of Compressive and Flexural Strength of a Novel Nanohybrid Composite Resin (Spident EsCom100) and an Established Nanohybrid Composite (Tetric N-Ceram): An In Vitro Study

**DOI:** 10.7759/cureus.115626

**Published:** 2026-09-01

**Authors:** Vipra Yadav, Leena H Jobanputra, Bhargav Patel, Shubhangi Ramteke, Mital Prajapati, Anupama Abhilasha Murmu

**Affiliations:** 1 Department of Conservative Dentistry and Endodontics, Government Dental College and Hospital, Jamnagar, IND; 2 Department of Community Medicine, Netaji Subhas Medical College and Hospital, Adityapur, IND

**Keywords:** compressive strength, flexural strength, nanohybrid composite, resin composite, spident escom100, tetric n-ceram, universal testing machine

## Abstract

Background and objective

Resin-based composites are widely used in restorative dentistry due to their esthetics and mechanical performance. However, variations in composition can influence their strength and clinical durability. This study aimed to comparatively evaluate the compressive and flexural strengths of the novel nanohybrid composite Spident EsCom100 (Spident Co., Incheon, South Korea) and the established nanohybrid composite Tetric N-Ceram (Ivoclar, Schaan, Liechtenstein).

Materials and methods

A total of 80 specimens were prepared and divided into two groups. We evaluated compressive strength using cylindrical specimens (n = 20) measuring 5 mm × 5 mm, and flexural strength using rectangular specimens (n = 20) measuring 25 mm × 2 mm × 2 mm in a three-point bending test. Testing was performed using a universal testing machine. Data were analyzed using an independent-samples t-test (p < 0.05).

Results

The mean compressive strength of Spident EsCom100 and Tetric N-Ceram was 116.21 ± 38.64 MPa and 116.47 ± 33.22 MPa, respectively, with no statistically significant difference (t = 0.023, p = 0.981). The mean flexural strength values were 82.98 ± 20.30 MPa for Spident EsCom100 and 84.13 ± 13.56 MPa for Tetric N-Ceram, which was also not statistically significant (t = 0.211, p = 0.828).

Conclusions

Both Spident EsCom100 and Tetric N-Ceram demonstrated comparable compressive and flexural strength values under the standardized laboratory conditions employed in this in vitro study, indicating satisfactory mechanical performance for restorative clinical applications.

## Introduction

Resin-based composites have undergone substantial evolution over the years, progressing from conventional macrofilled materials to microfilled, hybrid, microhybrid, and nanocomposite formulations [1]. Owing to their excellent esthetics, conservative tooth preparation, and versatile clinical applications, they have become the material of choice for direct restorative procedures [2]. Although significant improvements have been achieved in their physical, mechanical, and esthetic properties, the fundamental dimethacrylate-based resin matrix has remained largely unchanged since Bowen introduced it in the 1960s [3]. Consequently, most advancements in composite technology have been driven by innovations in filler systems, including modifications in filler size, morphology, distribution, and loading. These developments have significantly enhanced the strength, wear resistance, polishability, and clinical longevity of modern resin composites [4,5].

Recent advances in dental material science have led to the incorporation of nanotechnology, which involves the manipulation of materials at the nanoscale (0.1-100 nm) to enhance their physical, mechanical, and optical properties [6]. This has resulted in the development of nanohybrid resin composites, which combine nano-sized fillers with conventional filler particles to optimize both strength and esthetics [7]. Compared with earlier composite formulations, nanohybrid composites exhibit improved compressive and flexural strength, fracture and wear resistance, reduced polymerization shrinkage, enhanced translucency, and superior polish retention [8]. These properties make them suitable for both anterior and posterior restorations [9]. Since restorative materials are routinely exposed to complex compressive, tensile, and flexural forces during mastication, the mechanical performance of composite resins is critical for their long-term clinical success [10]. In this regard, compressive strength reflects the material's ability to withstand occlusal loads, while flexural strength evaluates its resistance to deformation and fracture under bending stresses [4].

Tetric N-Ceram (Ivoclar, Schaan, Liechtenstein) is a light-cured nanohybrid composite resin consisting of a dimethacrylate-based organic matrix incorporating Bis-GMA, UDMA, and TEGDMA monomers. Its inorganic phase comprises barium glass, ytterbium trifluoride, and mixed oxide fillers, along with prepolymerized filler particles. According to manufacturer specifications, the material exhibits a filler loading of approximately 75-77 wt% (53-55 vol%), with a wide particle size distribution ranging from nanoscale fillers to microhybrid particles, which collectively contribute to its well-documented mechanical strength and clinical performance.

Spident EsCom100 (Spident Co., Incheon, South Korea) is a recently introduced nanohybrid composite resin designed for direct restorative applications in both anterior and posterior teeth. It is a light-cured, dimethacrylate-based material containing silanated inorganic fillers intended to improve mechanical properties and wear resistance. The manufacturer claims advantageous properties, including high compressive strength, reduced polymerization shrinkage, extended working time, and satisfactory esthetic integration with natural tooth structure. However, much of the available evidence is based on manufacturer-reported data, with limited independent studies assessing its mechanical behavior.

Therefore, there is a need for further investigation to comparatively assess the mechanical properties of this novel nanohybrid composite and an established nanohybrid composite system and provide evidence-based guidance for their selection in clinical practice. This study aimed to comparatively evaluate the compressive strength and flexural strength of Spident EsCom100 and Tetric N-Ceram composite resins under standardized laboratory conditions. The null hypothesis tested was that there is no statistically significant difference in compressive strength or flexural strength between Spident EsCom100 and Tetric N-Ceram.

## Materials and methods

This in vitro study was conducted in the Department of Conservative Dentistry and Endodontics, Government Dental College and Hospital, Jamnagar. Two nanohybrid composite resins were evaluated: Group A - Spident EsCom100; Group B - Tetric N-Ceram.

Sample size calculation was performed using G*Power software (Version 3.1.9.4, Heinrich Heine University, Düsseldorf, Germany) based on data reported by Ramdas et al. on the compressive and flexural strength of composite restorative materials [11]. Using a significance level (α) of 0.05, a statistical power of 80% (1 − β = 0.80), and an effect size of 0.81, the minimum required sample size was calculated as 16 specimens per subgroup. To improve statistical robustness and compensate for potential specimen fabrication defects and testing-related variability, the sample size was increased to 20 specimens per subgroup.

Accordingly, a total of 80 specimens were included in the study, with 40 specimens allocated to each material group. Each group was further subdivided into Subgroup A (compressive strength testing; n = 20) and Subgroup B (flexural strength testing; n = 20), resulting in 20 specimens per material for each mechanical property evaluated.

Specimen fabrication

Standardized cylindrical (5 mm × 5 mm) and rectangular (25 mm × 2 mm × 2 mm) specimens were fabricated using a three-step procedure. Initially, master specimens of the required dimensions were designed and fabricated using 3D printing technology. Negative replicas of these master specimens were then prepared using addition silicone putty material to obtain standardized moulds for specimen preparation (Figure 1).

**Figure 1:**
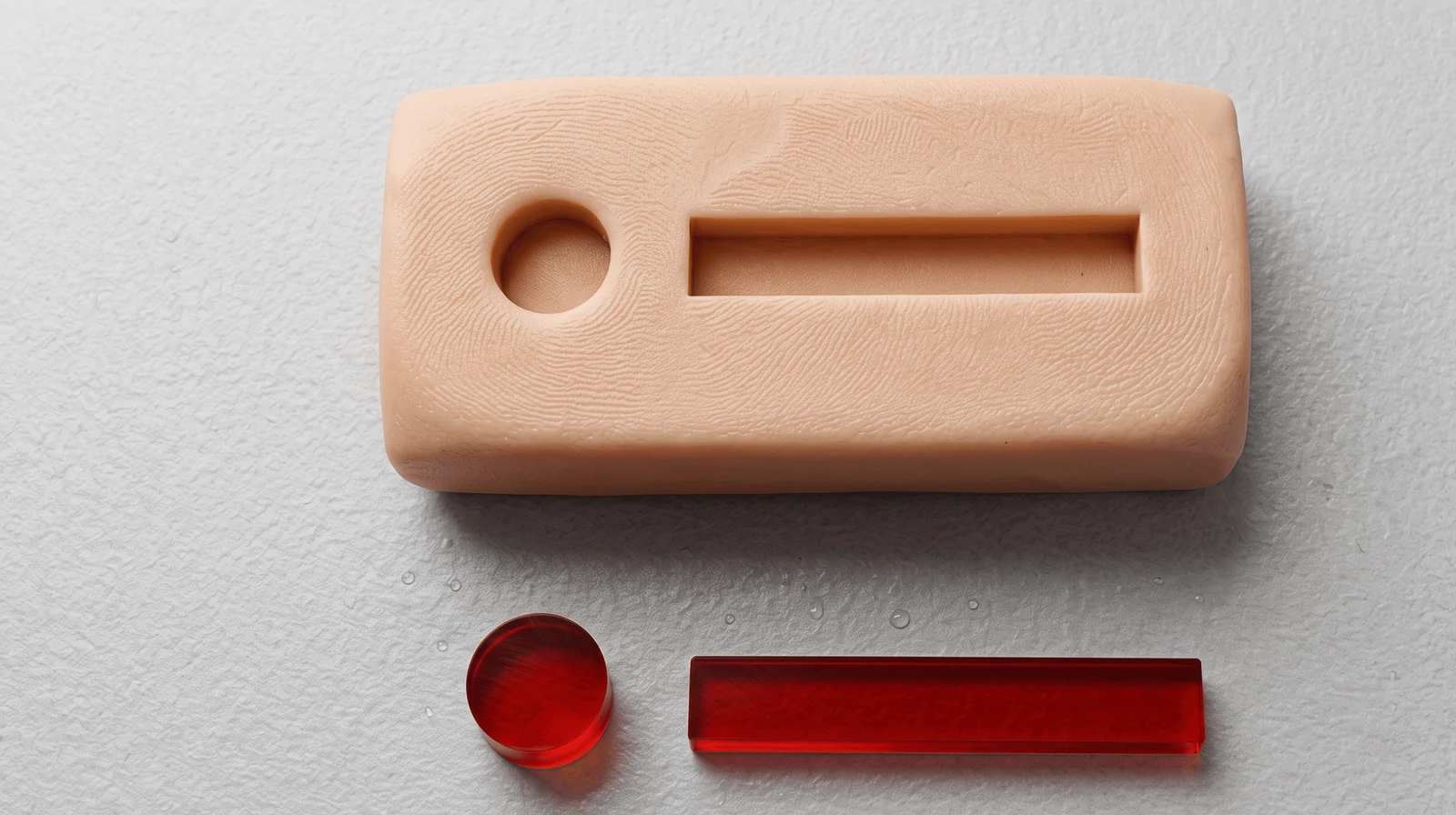
Fabrication of a putty index mould and the corresponding 3D-printed specimen

The composite resin was packed into the moulds using an incremental technique, with each increment not exceeding 2 mm in thickness. Each layer was light-cured for 40 seconds using an LED curing unit (Woodpecker RTA Mini S; intensity ≥ 1000 mW/cm²; wavelength 430-480 nm). A Mylar strip was placed over the composite surface during curing to obtain a smooth, standardized surface free of an oxygen-inhibition layer. After removal from the moulds, all specimens were post-cured from all directions for 60 seconds to ensure complete polymerization. The specimens were subsequently stored in distilled water at 37°C for 24 hours prior to mechanical testing in accordance with standardized laboratory protocols.

Testing procedures

All specimens were subjected to mechanical testing using a universal testing machine (ACME Engineers, Pune, India).

Compressive Strength Test

Cylindrical specimens (5 mm diameter × 5 mm height) were placed with their long axis parallel to the direction of the applied load. A constant crosshead speed of 1 mm/min was maintained until fracture. The maximum load at fracture (P) was recorded in Newtons (N), and compressive strength (CS) was calculated using the formula:

CS (MPa) = P / (πr²)

Where P is the fracture load (N), r is the radius of the specimen (mm), π = 3.1416, and MPa = megapascal (Figure 2).

**Figure 2:**
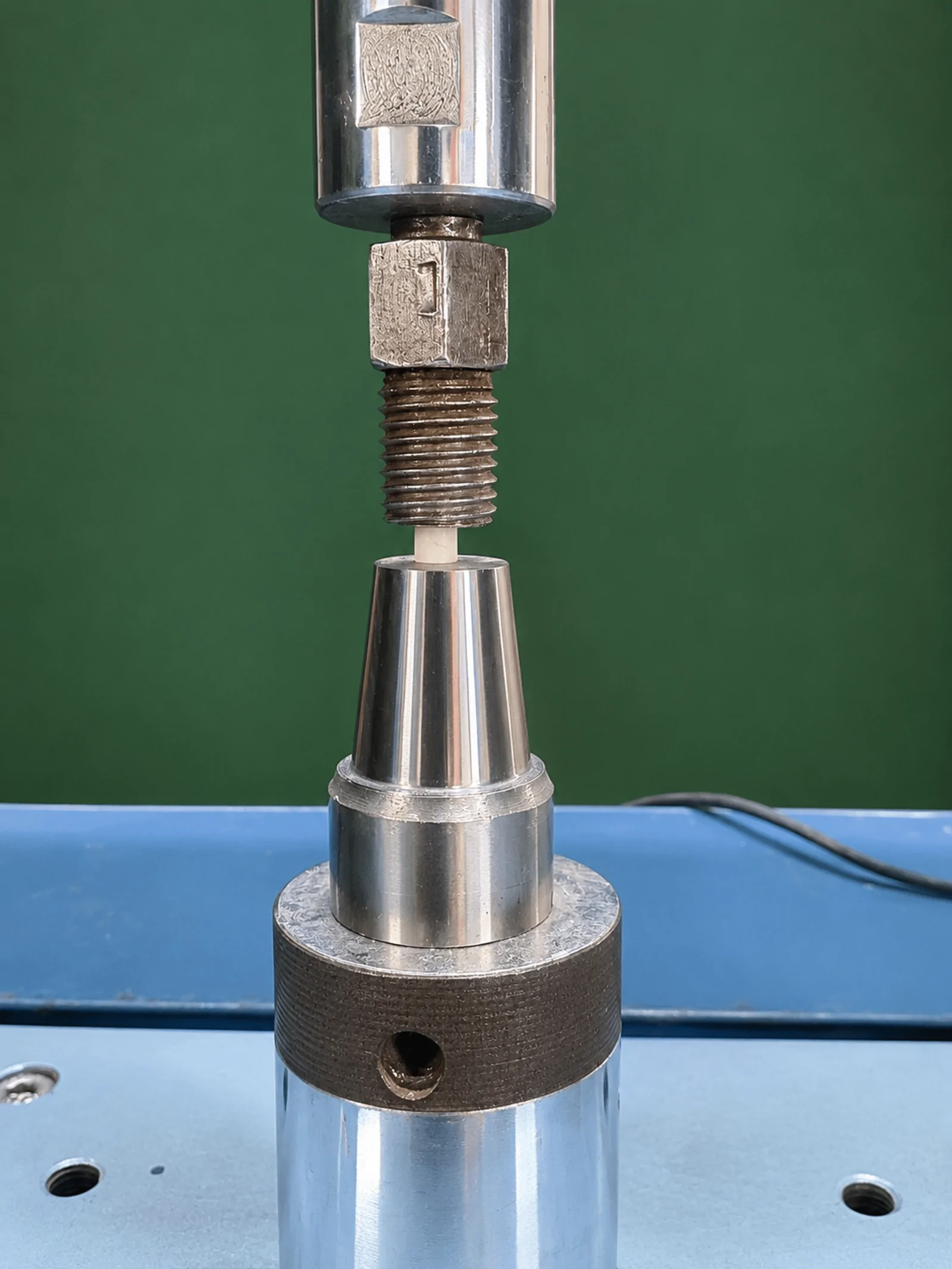
Compressive strength testing setup showing a cylindrical specimen loaded under a universal testing machine

Flexural Strength Test

Flexural strength was determined using a three-point bending test in accordance with the ISO 4049:2019 specification [12]. Bar-shaped specimens measuring 25 mm × 2 mm × 2 mm were fabricated. Each specimen was placed on a universal testing machine with a support span of 20 mm, and load was applied at the centre at a crosshead speed of 1 mm/min until fracture (Figure 3).

**Figure 3:**
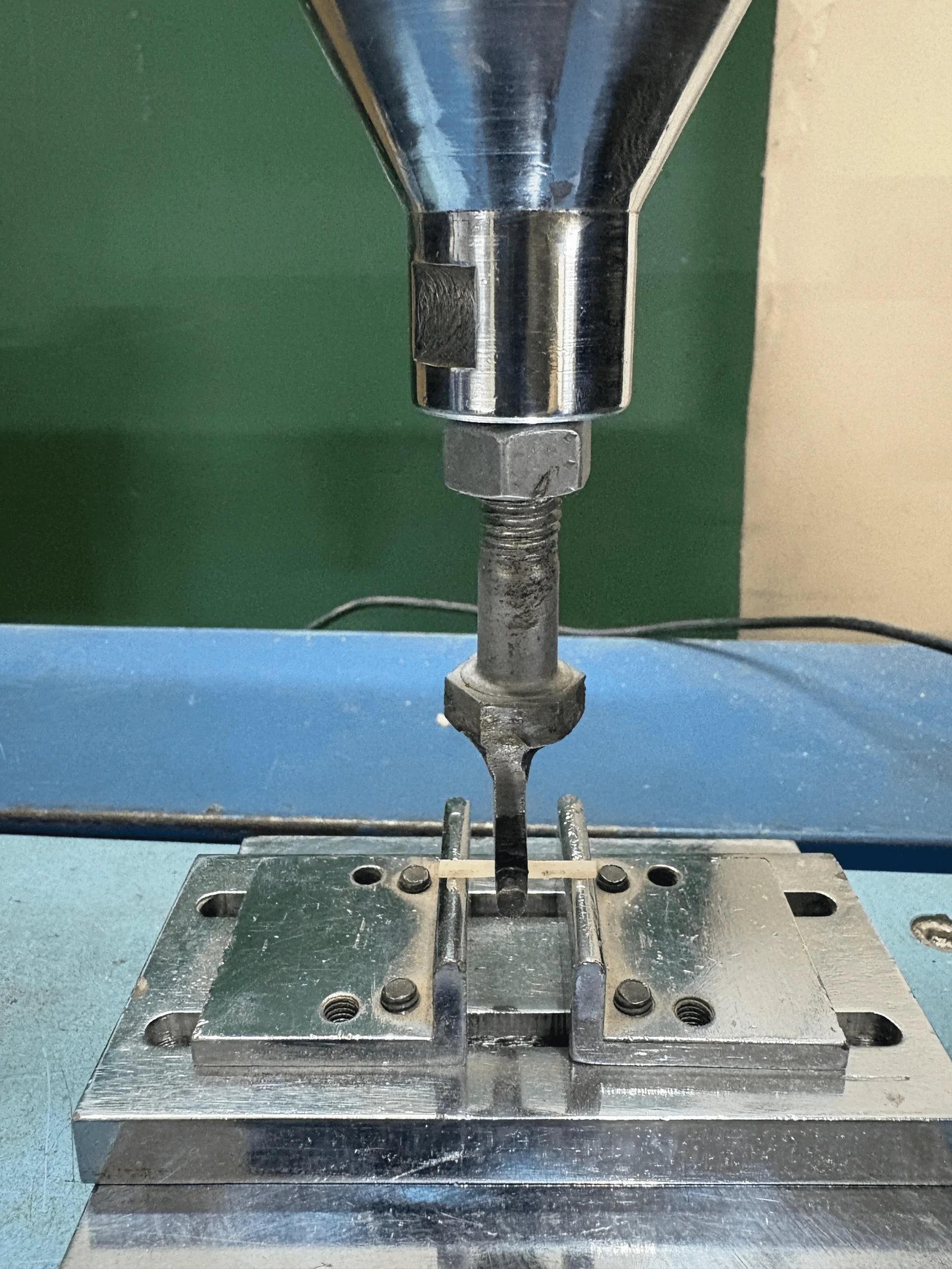
Flexural strength testing setup showing a three-point bending arrangement for a rectangular bar specimen

The flexural strength (FS) was calculated using the formula:

FS (MPa) = 3PL / (2bd²)

Where P = load at fracture (N), L = span length (20 mm), b = specimen width (mm), d = specimen thickness (mm), and MPa = megapascal.

Statistical analysis

Statistical analyses were conducted using IBM SPSS Statistics for Windows, Version 29.0 (IBM Corp., Armonk, NY). The normality of data distribution was evaluated using the Shapiro-Wilk test. Since all variables satisfied the assumption of normality (p > 0.05), comparisons between the two independent groups (n = 20 per group) were performed using a two-tailed independent-samples t-test. Results were expressed as mean ± standard deviation (SD), and a p-value < 0.05 was considered statistically significant.

## Results

The mean compressive strength values were 116.21 ± 38.64 MPa for Spident EsCom100 (Group A) and 116.47 ± 33.22 MPa for Tetric N-Ceram (Group B), with no statistically significant difference between the groups (t = 0.023, p = 0.981). Similarly, the mean flexural strength values were 82.98 ± 20.30 MPa and 84.13 ± 13.56 MPa for Group A and Group B, respectively (t = 0.211, p = 0.828). No statistically significant differences were observed between Spident EsCom100 and Tetric N-Ceram for either compressive or flexural strength (Table 1).

**Table 1:** Comparison of mean compressive and flexural strength values between Spident EsCom100 (Group A) and Tetric N-Ceram (Group B) Statistical test: independent-samples t-test (two-tailed); df = 38; significance set at p < 0.05 SD: standard deviation

Test parameter	Group A (Spident EsCom100) (n = 20), mean ± SD	Group B (Tetric N-Ceram) (n = 20), mean ± SD	t-value	P-value
Mean compressive strength (MPa)	116.21 ± 38.64	116.47 ± 33.22	0.023	0.981
Mean flexural strength (MPa)	82.98 ± 20.30	84.13 ± 13.56	0.211	0.828

## Discussion

Resin-based composites are extensively used in contemporary restorative dentistry because they combine excellent esthetics with satisfactory mechanical performance. Contemporary nanohybrid composites have been developed to improve the mechanical and physical properties of restorative materials while maintaining excellent esthetics [13]. Among the various factors influencing the clinical longevity of composite restorations, compressive strength and flexural strength are particularly important because restorations are continuously exposed to complex compressive, tensile, and bending forces during mastication [14]. Therefore, evaluation of these properties provides valuable information regarding the ability of restorative materials to withstand functional stresses and maintain structural integrity over time [15].

The present study compared the compressive and flexural strengths of two nanohybrid composite resins, Spident EsCom100 and Tetric N-Ceram. The results demonstrated no statistically significant differences between the materials in either compressive strength or flexural strength, thereby supporting the null hypothesis. These findings indicate that both materials exhibit comparable mechanical performance under the standardized experimental conditions employed in this study (Table 1).

The comparable mechanical performance observed in the present study may be attributed to the nanohybrid design shared by both materials [6,16]. Nanohybrid composites incorporate nano-sized fillers together with conventional filler particles, enabling improved filler packing and effective reinforcement of the resin matrix [17]. The mechanical behavior of these materials is largely determined by their microstructural characteristics, including filler content, filler distribution, and the quality of the filler-matrix interface, which collectively influence load transfer and resistance to structural failure [18]. Previous studies have demonstrated that optimized filler loading and strong filler-matrix interactions contribute significantly to the strength and durability of resin-based composites. Furthermore, advances in filler technology and composite architecture have been associated with improved mechanical reliability and enhanced resistance to functional stresses in contemporary nanohybrid restorative materials [19].

Although Tetric N-Ceram demonstrated slightly higher mean compressive and flexural strength values than Spident EsCom100, these differences were not statistically significant. The slight differences observed between the materials may be attributed to variations in filler composition, filler content, particle size distribution, and resin matrix characteristics. However, under the testing conditions employed, these compositional differences did not result in a measurable difference in overall mechanical performance.

The mechanical properties observed in the present study were consistent with those reported for contemporary nanohybrid resin composites by Ramdas et al., Păstrav et al., Ali et al., and Jayanthi et al. when evaluated using comparable testing protocols [11,20-22]. Variations in reported compressive and flexural strength values across studies may be related not only to differences in material composition but also to methodological factors such as specimen preparation, curing conditions, storage protocols, and the mechanical testing modality employed. These factors are known to influence the measured performance of resin-based composites and may contribute to the variability reported in the literature [23].

The reliability of the present findings was enhanced through strict standardization of specimen fabrication, curing procedures, storage conditions, and mechanical testing protocols. Furthermore, flexural strength testing was performed using the three-point bending method in accordance with ISO 4049, which remains the internationally accepted standard for evaluating the bending behavior of polymer-based restorative materials [12,23].

From a clinical perspective, the absence of significant differences between Spident EsCom100 and Tetric N-Ceram suggests that the newer material possesses mechanical properties comparable to those of an established nanohybrid composite resin. This finding is particularly relevant because adequate compressive and flexural strength are essential for the long-term success of direct restorations in stress-bearing areas. Considering its comparable mechanical performance and potential economic advantage, Spident EsCom100 may be regarded as a promising alternative for direct restorative applications. Nevertheless, additional investigations evaluating wear resistance, fracture toughness, polymerization shrinkage, color stability, and long-term aging behavior are required before definitive clinical recommendations can be established.

Limitations and future directions

In vitro testing cannot fully simulate the oral environment, which includes temperature changes, salivary enzymes, and both functional and parafunctional forces. Thermocycling and long-term aging are important for a more comprehensive assessment of material performance. Future studies should incorporate these factors, along with larger, multicenter sample sets, to more accurately evaluate the long-term clinical behavior.

## Conclusions

Within the limitations of this in vitro study, the novel nanohybrid composite Spident EsCom100 demonstrated compressive and flexural strength comparable to those of the established nanohybrid composite Tetric N-Ceram, indicating satisfactory mechanical performance for restorative clinical applications. This finding suggests that Spident EsCom100 may serve as a viable alternative to established nanohybrid composites in direct restorative procedures. Nevertheless, further studies incorporating thermocycling, long-term aging, and additional mechanical and clinical parameters are required before definitive clinical recommendations can be made.
